# Promising clinical outcome after body gamma knife radiotherapy for mediastinal follicular dendritic cell sarcoma with thoracic spine invasion and iliac metastasis: A case report and literature review

**DOI:** 10.3389/fonc.2022.919644

**Published:** 2022-09-16

**Authors:** Annan Hu, Ting Chen, Jian Dong

**Affiliations:** ^1^ Department of Orthopaedic Surgery, Zhongshan Hospital, Fudan University, Shanghai, China; ^2^ Department of Radiotherapy, People’s Liberation Army of China (PLA) Naval Medical Center, Naval Medical University, Shanghai, China

**Keywords:** body gamma knife, stereotactic body radiotherapy, follicular dendritic cell sarcoma, pathological complete response, separation surgery

## Abstract

**Background:**

Follicular dendritic cell sarcoma (FDCS) is a rare type of intermediate grade tumor. Mediastinal FDCS with spinal invasion has not been well described. The treatment options include surgical resection and radiation therapy. The body gamma knife is a stereotactic body radiotherapy (SBRT) technology that is widely used in China. The pathological evaluation of a bone lesion after a body gamma knife procedure has not been reported. Here, we report a case of a patient with FDCS with thoracic spine invasion and iliac metastasis treated with surgery and body gamma knife.

**Case summary:**

A 36-year-old male patient was hospitalized at Zhongshan Hospital, Fudan University, due to a gradually aggravated pain on the lateral side of the left scapula for 6 months. Imaging examination showed neoplastic lesions on the left side of C7-T2 invading the vertebral body of T1, T2, and caput costae of the second rib and suspected metastasis in the left ilium. FDCS was diagnosed after performing a computed tomography (CT)-guided core needle biopsy, and the thoracic lesion was surgically resected. The body gamma knife was used as an adjuvant radiotherapy for the thoracic lesion and a primary therapy for the left ilium lesion. Iliac bone lesion resection was performed at Zhongshan Hospital, Fudan University, 10 weeks after RT. Compared with the biopsy report, the body gamma knife treatment resulted in a pathological complete response (PCR). The magnetic resonance imaging (MRI) examinations showed stable disease of the thoracic lesion after body gamma knife radiosurgery.

**Conclusion:**

This case report describes the treatment of mediastinal FDCS with thoracic spinal invasion and iliac metastasis. The promising outcome suggests that separation surgery is an effective treatment option for mediastinal FDCS with spinal column invasion. It also demonstrates the application prospects of the body gamma knife treatment in malignant lesions of the axial bones.

## Introduction

Follicular dendritic cell sarcoma (FDCS) was first described by Monda et al. in 1986 (1). FDCS most commonly presents as a slow-growing mass in the cervical and axillary lymph nodes (2, 3). Some studies reported more extranodal cases, which may be due to referral bias (4, 5). Among all the reported FDCS cases that occurred at the extranodal sites, only a few involved the mediastinum, and none of these case reports adequately described spinal column involvement and concurrent metastasis (4, 6). There is no standard treatment for FDCS. A combined modality approach consisting of surgery, radiotherapy (RT), and/or chemotherapy is most commonly used (6, 7). Jain et al. analyzed 66 cases and concluded that aggressive local treatment with surgery and adjuvant RT improved local control (5). A few studies have also reported the effectiveness of targeted therapy and immunotherapy (8, 9).

As one of the stereotactic body radiotherapy (SBRT) systems, the body gamma knife uses 30 or 18 Co^60^ as the radiation source to release high-dose gamma rays that focus on a target area (10). It has sharp dose gradients such that the normal tissue around the target area receives a very low dose of radiation (11). The body gamma knife has been used in the treatment of several types of solid tumors, including lung cancer, pancreatic carcinoma, and liver cancer, in China (10, 12, 13). No study has reported the application of a body gamma knife treatment in FDSC or in axial bones. After body gamma knife RT, patients generally no longer undergo surgical resection of the target area; thus, it is difficult to perform a pathological evaluation for the body gamma knife treatment. Herein, we report a case of a patient with mediastinal FDCS with thoracic spinal invasion and iliac metastasis. The thoracic lesion was successfully treated by decompression surgery and postoperative adjuvant body gamma knife RT. Iliac metastasis demonstrated a pathological complete response (PCR) after body gamma knife RT.

## Case description

**Figure 1:**
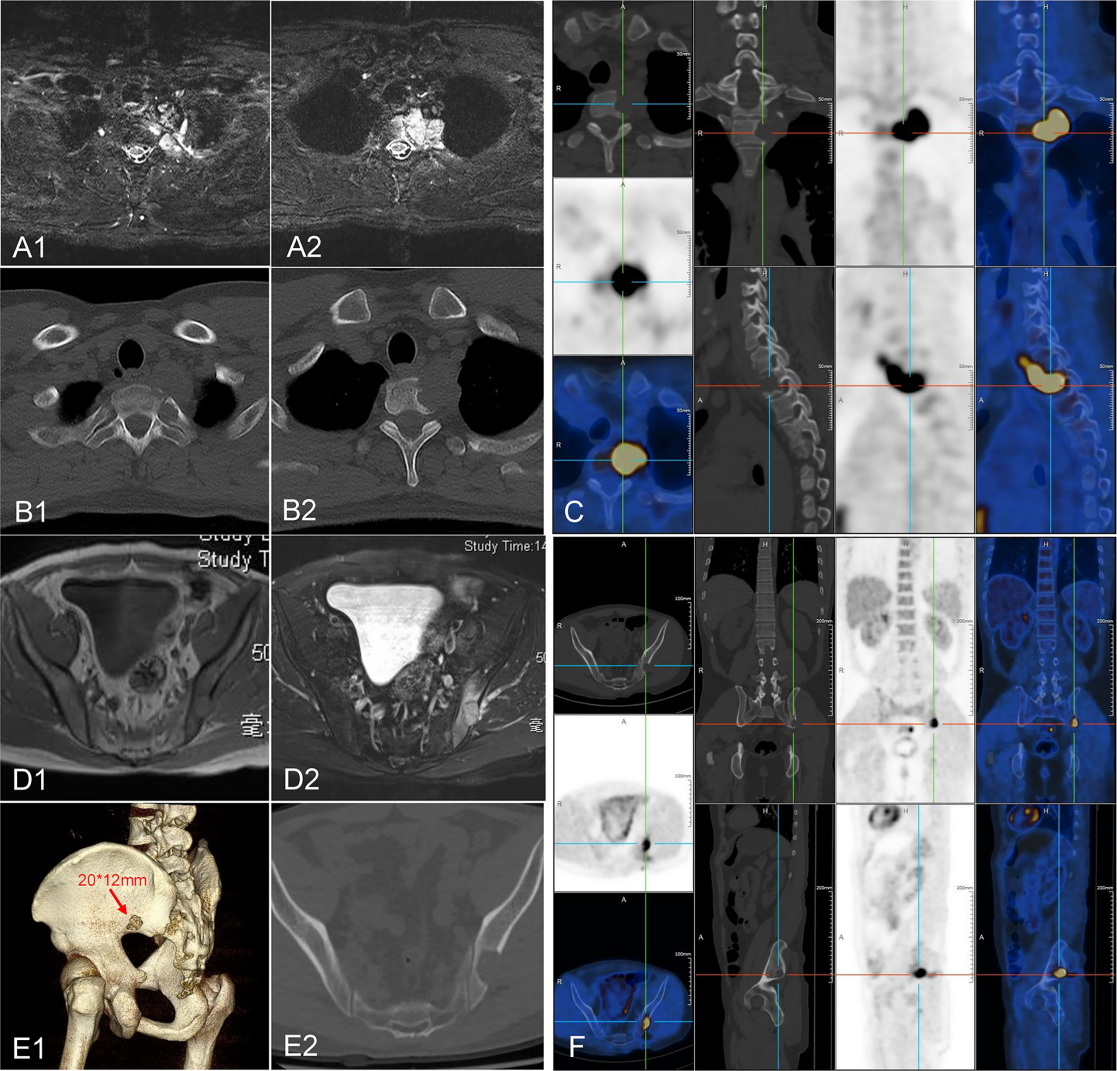
Image of the neoplastic lesions. **(A–C)** Neoplastic lesions found on the left side of C7-T2 invading the vertebrae body of T1, T2, and caput costae of the second rib. **(D–F)** A suspected metastasis was found in the left ilium.

**Figure 2:**
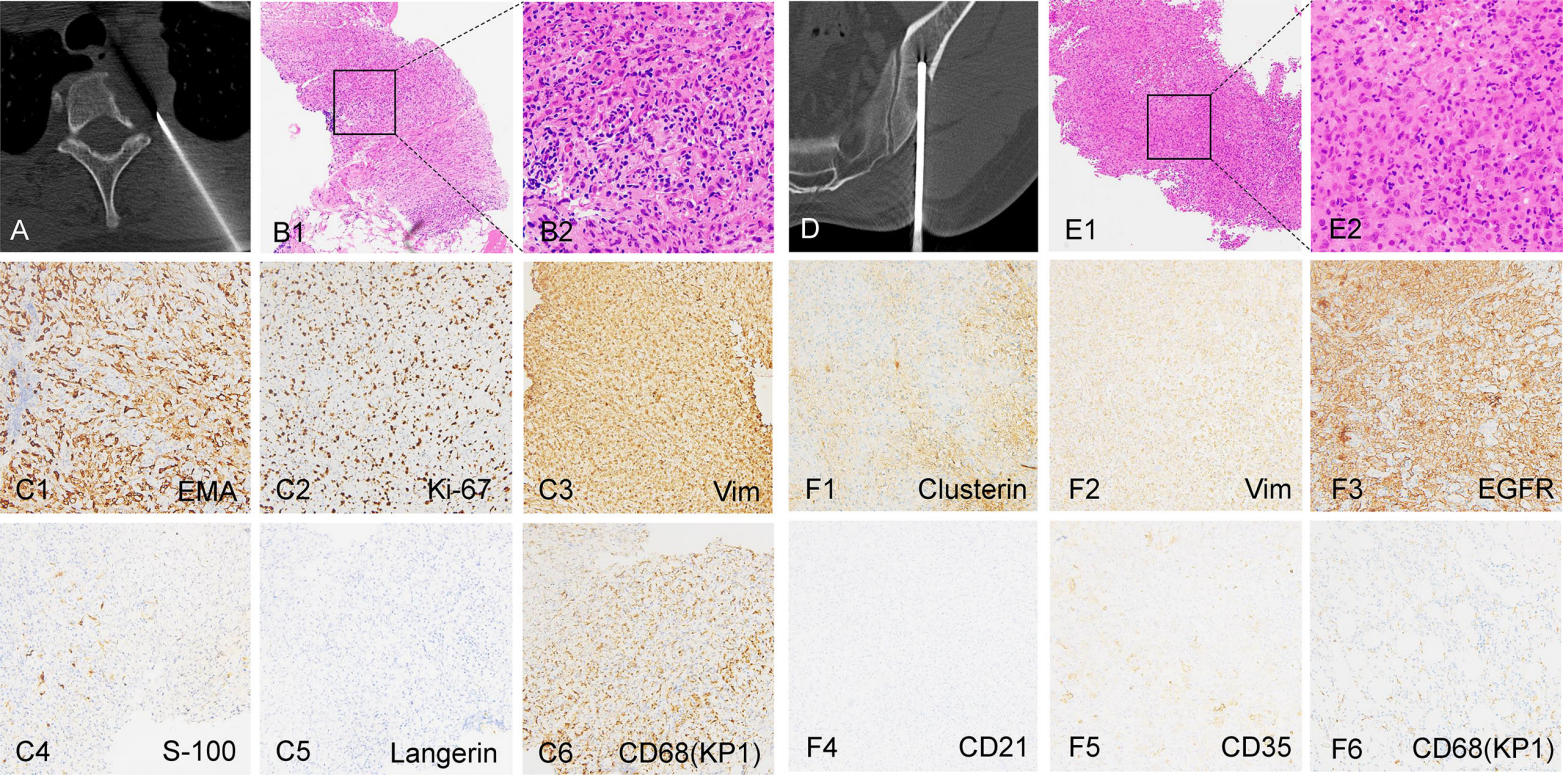
Biopsy and staining images of the tumors. **(A, B)** CT-guided core needle biopsy and the H&E staining images of the thoracic lesion. (C1-C6) Immunohistochemical staining of the thoracic lesion. **(D, E)** CT-guided core needle biopsy and the H&E staining images of the left ilium lesion. (F1-F6) Immunohistochemical staining of the left ilium lesion. B1, E1: 100×; B2, E2: 400×; **(C, F)**: 200×.

A 36-year-old male patient was hospitalized due to complaints of a gradually aggravated pain on the lateral side of the left scapula for 6 months. Imaging examination showed that the neoplastic lesions on the left side of C7-T2 invaded the vertebral body of T1, T2, and caput costae of the second rib (**Figures 1A–C**). Tumor metastasis in the left ilium was suspected (**Figures 1D–F**). A computed tomography (CT)-guided core needle biopsy was performed to obtain the tissue samples of the paravertebral lesion in T2 and the lesion in the left ilium. The pathological results confirmed the diagnosis of FDCS. Diffuse small spindle cells were found in the hematoxylin and eosin (H&E) staining of both paravertebral (**Figures 2A, B**) and iliac (**Figures 2D, E**) lesions. Immunohistochemical staining of the paravertebral sample was positive for cytokeratin (CK){pan} and vimentin; partially positive for cluster of differentiation 68 (CD68){KP1} and epithelial membrane antigen (EMA); slightly positive for S100 and CD34; and negative for SRY-box transcription factor 10 (Sox10), Langerin, thyroid transcription factor 1 (TTF-1), and prostatic specific acid phosphatase (PSAP) (**Figure 2C**). Immunohistochemical staining of the iliac sample was positive for CK{pan}, vimentin, CD68{KP1}, and epidermal growth factor receptor (EGFR); partially positive for Clusterin; slightly positive for S100, CD35, and CD20; and negative for CD21, Sox10, Langerin, and CXC chemokine ligand 13 (CXCL13) (**Figure 2F**).

**Figure 3:**
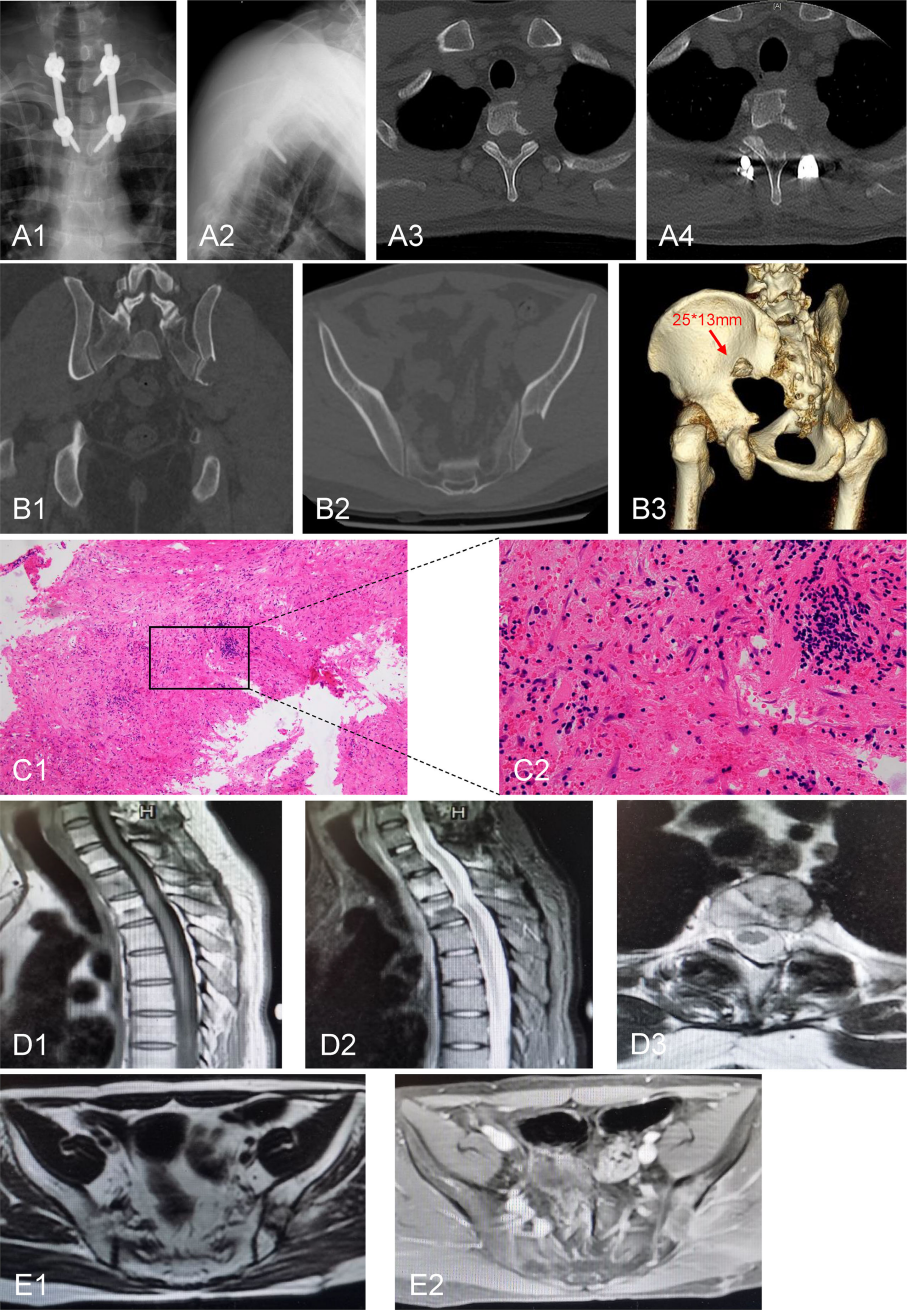
Postoperative radiological and pathological images. **(A1, 2)** Pedicle screw internal fixation was performed at the level of T1-T3. **(A3, 4)** Postoperative CT scan showed that the left lamina and the facet joint of T1 and T2 were resected. **(B1-3)** CT examination of the iliac bone showed that the lesion size was slightly larger than that before the 2-month postoperative follow-up. **(C1, 2)** H&E staining of iliac bone lesion. **(D1-3)** Thoracic MRI examination showed no significant enlargement of the lesion after 15 months of follow-up. **(E1, 2)** Pelvic MRI examination showed absence of recurrence of the iliac lesion after 15 months of follow-up. C1: 100×; C2: 400×.

Tumor resection and nerve root decompression were performed as treatment for thoracic disease. A midline incision was made, and the lamina and facet joints of T1–T3 were exposed. Four pedicle screws were implanted in T1 and T3 (**Figures 3A1, 2**). The left lamina and facet joint of T1 and T2 were resected (**Figures 3A3, 4**). The left second costovertebral joint and the second rib head were also resected. Transpedicular curettage was performed to ensure sufficient neural decompression of the tumor, providing a safe target volume for radiation.

The body gamma knife was used as a radical treatment for iliac tumor and as an adjuvant treatment for thoracic disease. Body gamma knife planning and delivery were similar to those reported in previous studies (10, 12, 13). The patient was placed in a supine position and immobilized using a vacuum negative pressure bag and a body frame bed, allowing to breathe naturally. Markings were made on the four areas of the body that will receive the radiation to ensure reproducible body position. The CT images were transferred to the OPEN body gamma knife treatment planning system. A local bone destruction lesion was found at the posterior lower edge of the left ilium (near the sacroiliac joint) with clear boundaries and soft tissue density. The treatment target area was determined in the bone window on the CT image using the body gamma knife treatment planning system (window width, 1,000 HU; window level, 300 HU). The gross target volume (GTV) was delineated according to the lesion identified in the bone window. The clinical target volume (CTV) was generated by extending the GTV by 5 mm in all directions. The planning target volume (PTV) was generated by extending the CTV by 5 mm in all directions. The 50% radiation dose covered 100% of the PTV, the 60% dose covered 100% of the CTV, and the 70% dose covered 100% of the GTV. For the iliac lesion, the prescription dose for the PTV, CTV, and GTV margins were 40, 48, and 56 Gy in 10 fractions, respectively. The highest physical dose delivered at the center of the target area was 80 Gy. The biological effective dose (BED) of the RT was calculated using a linear quadratic (LQ) model, assuming an α/β ratio of 10. The BEDs at the margins of PTV, CTV, and GTV were 56, 71.04, and 87.36 Gy, respectively, and the highest BED delivered at the center of the target area was 144 Gy. For the thoracic lesion, the prescription doses for PTV, CTV, and GTV margins were 36, 43.2, and 50.4 Gy in 12 fractions, respectively. The highest physical dose delivered at the center of the target area was 72 Gy. The BEDs delivered to the margins of the PTV, CTV, and GTV were 46.8, 58.752, and 71.568 Gy, respectively, and the highest BED delivered at the center of the target area was 115.2 Gy. Radiotherapy target planning of thoracic and iliac lesions was presented in **Supplementary Figures 1, 2**. The treatment process proceeded smoothly, the patient had no complaints, and no abnormal findings were found on blood tests after RT.

**Figure 4:**
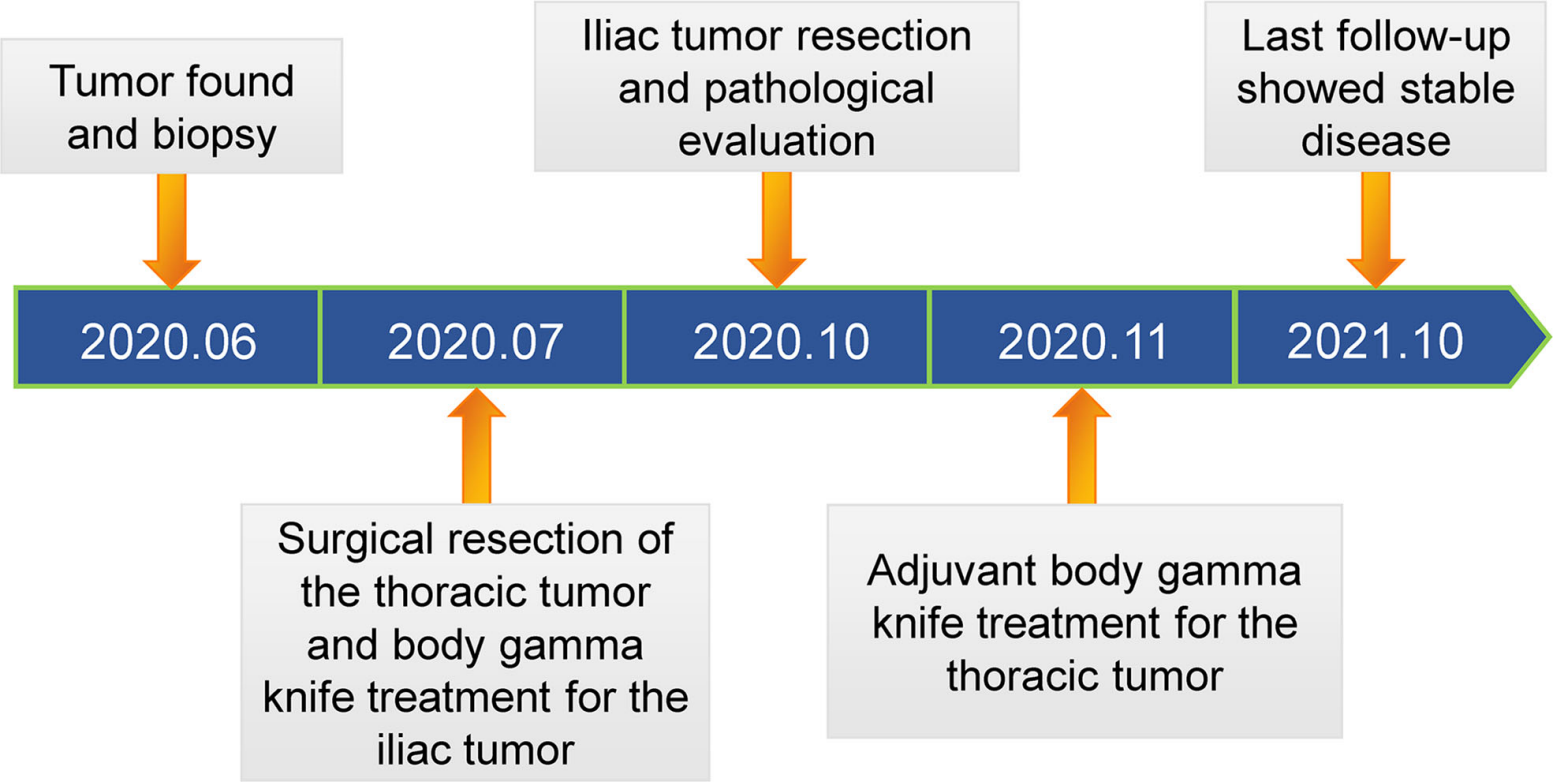
Timeline of the occurrence of major clinical events during the treatment and follow-up.

The patient did not receive systemic treatment or other local control treatments after the body gamma knife treatment. CT examination of the iliac bone showed that the lesion size was slightly larger than that before the 2-month postoperative follow-up (**Figures 3B1–3**). Thus, iliac bone lesion resection was performed 10 weeks after RT. The pathological examination of the left iliac bone tumor suggested a small amount of bone tissue, fibrous tissue hyperplasia and hemorrhage, myofibroblast reaction, lymphocyte and plasma cell infiltration, a small amount of necrosis, and tissue cell deposition, while no tumor tissue was observed (**Figures 3C1, 2**). Thus, the body gamma knife treatment resulted in PCR. The patient was followed up for 1 year, and the VAS score for back pain reduced from 8 at preoperatively to 1 at the last follow-up. Thoracic and pelvic magnetic resonance imaging (MRI) examinations showed no significant enlargement or recurrence of the tumors after 15 months of follow-up (**Figures 3D, E**). The timeline of major clinical events during treatment and follow-up is shown in **Figure 4**.

## Discussion

FDCS is a type of intermediate grade tumors originating from follicular dendritic cells in the germinal center of lymph nodes. It was first reported by Monda et al. (1) in 1986. The biological behavior of FDCS is similar to that of intermediate-grade soft-tissue sarcomas. The head, neck, and mediastinal FDCS cases have good prognosis, whereas a minority of cases develop local recurrence (12.5%–28.1%) and metastases (13.2%–27.2%) (7, 14, 15). The most common metastatic sites are the lung and liver; other metastatic sites include the adrenal gland, rib, vertebral body, and iliac bone (14). Metastasis of the iliac bone was observed in the present case. The reported age of onset is 9–79 years (average: 46 years) (14). FDCS tends to grow slowly and is usually asymptomatic (16). The lesions usually are hypermetabolic and present as avid spots on positron emission tomography (PET) images (3, 17). There is a subtype of FDCS that almost exclusively occurs in the liver and spleen. It is different from the FDCS that occurs in other parts. The World Health Organization (WHO) has recognized a distinct entity named inflammatory pseudotumor (IPT) like FDCS (18). It is speculated that IPT like FDCS is related to the Epstein–Barr virus (EBV) infection and is more common in women (19). The immunohistochemical results support the diagnosis of FDCS to a large extent. Normal FDC-associated markers, such as CD21, CD23, CD35, and CXCL13, are widely used. Multiple markers are often used owing to the frequent loss of antigens (20, 21). Clusterin is a sensitive and specific marker for FDCS (22). In addition, vimentin, EGFR, EMA, S-100, and CD68 were used to confirm the diagnosis (20). Immunohistochemical staining was not typical in the present case. CD35 was slightly positive and CD21 was negative. Morphologically, the nuclei appeared to be short fusiform to oval, some of the nuclei showed vacuolar-like changes, some showed small nucleoli, the cytoplasm was lightly stained, and the interstitium was scattered with lymphocytic infiltration. Based on the morphological and immunohistochemical findings, the diagnosis of FDCS was made.

To date, there is no standard therapeutic protocol for FDCS, and different approaches have been applied, including surgery, radiotherapy, and chemotherapy. Surgical resection is the primary treatment for FDCS, and the incidence of recurrence is relatively high (23). Although adjuvant radiotherapy or systemic therapy has not caused a significant improvement in survival (24), it can be applied in patients with advanced cases (7). Patients with extranodal disease experience poor outcomes and often require combined therapy (5). Conry and Gupta reported cases in which gemcitabine and docetaxel were used as treatment for metastatic FDCS, which showed promising outcomes (25, 26). FDCS also responded to some commonly used targeted agents and immune checkpoint inhibitors. Several FDCS case reports have shown that patients may benefit from kinase inhibitors, including pazopanib, sorafenib, and sirolimus (6, 9). The programmed cell death protein 1 (PD-1) antibody is a potential therapeutic option for patients with refractory FDCS (8, 27). Localized tumors are associated with better prognosis (6). Young age, large tumor size, high mitotic index, abdominal involvement, coagulative necrosis, and significant cellular atypia were associated with poor prognosis (7).

Considering that adjuvant radiotherapy and/or chemotherapy are often used for advanced FDCS, we introduced the concept of hybrid therapy in the treatment of this patient. Hybrid therapy consists of separation surgery and postoperative SBRT, which represents the evolution of treatment for spinal metastases (28). Separation surgery focuses on decompressing the spinal cord or nerves to achieve a safe margin for RT. This was an innovative attempt to this patient. Residual paraspinal tumors can be effectively treated with SBRT. SBRT involves the delivery of a high radiation dose within a shorter course, has obvious radiobiological advantages, lessens the risk of damage to the surrounding organs, and has less complications (29). Body gamma knife SBRT can be applied in a wide range of conditions and is widely used in China. It delivers a high radiation dose to the tumor focus and skin and generates an effect similar to that of scalpel resection, which is suitable for the treatment of patients in different positions. This highly focused SBRT technique has demonstrated satisfactory local control, reduced toxicity, and cost effectiveness in the treatment of non-small cell lung cancer (10, 30). Other initiatives have been carried out to explore the efficacy of the body gamma knife in other malignancies, such as pancreatic cancer (12). In this case, the 50% dose line was used as the prescribed dose line. The unique dose distribution and treatment pattern escalate the focused dose administered to the target area, that is, from the margin to the center, while the normal tissue outside the margin receives an extremely low dose, which is reasonable in radiobiology. Cao et al. (11) reported the dose distributions of five stereotactic body radiotherapies for pancreatic cancer. Gamma knife provided the highest mean and maximum dose to the PTV, excellent gradient index, and rapid dose fall-off. In addition, the body gamma knife is less expensive compared with other SBRT technologies, which is appealing especially in developing and underdeveloped countries (10). This patient did not receive adjuvant chemotherapy or targeted therapy. This is partly because there is no consensus on the optimal treatment recommendations for FDCS. On the other hand, adjuvant systemic therapy was not associated with improvement in the median or 5-year overall survival (OS) when compared to surgery alone (3, 6). In addition, performing the separation surgery has helped us to accumulate relatively more experience in SBRT, and thoracic and pelvic MRI showed stable disease after 15 months of follow-up. Based on the above considerations, this patient was treated with RT as adjuvant therapy for the thoracic lesion and as the primary treatment for the iliac metastasis. Values of α/β ratios in human individuals are rarely known, and there is no predictive assay for α/β for individual tumors. The use of α/β = 5, 10 and 15 Gy was recommended in most situations (31). An α/β ratio of 10 was applied in the calculation of BED in the present study.

In most cases, the effectiveness of RT is assessed though clinical evaluation, such as imaging and OS assessment, other than the gold standard histopathological examination. Biopsies may be used in a few cases to verify the treatment effect. A reliable pathological evaluation of malignant tumors of the body after gamma knife treatment has not yet been reported. In this case, mild edema was found in the target area, which was slightly enlarged on CT imaging at 2 months’ follow-up. Although this may be a reaction after RT, resection was performed as requested by the patient, and the whole tissue was obtained for evaluation. Pathological diagnoses before and after treatment were performed at the same hospital. After careful consultation with skilled experts, the consistency and authority of the diagnosis were ensured. This unique case showed that body gamma knife treatment can achieve not only CR but also PCR.

Some limitations of the body gamma knife should be recognized and improved. This procedure is insufficient in image guidance and localization for repeated patient positioning and the assessment for radiation dose assessment of the target area. The rapid development of medical imaging technique in recent years has enhanced the precision and efficiency of the body gamma knife.

## Conclusion

This case report describes the treatment of mediastinal FDCS with thoracic spinal invasion and iliac metastasis. The promising outcome suggests that separation surgery is an effective treatment option for mediastinal FDCS with spinal column invasion. It also demonstrates the application prospects of the body gamma knife treatment in malignant lesions of the axial bones.

## Data availability statement

The original contributions presented in the study are included in the article/**Supplementary Material**. Further inquiries can be directed to the corresponding authors.

## Ethics statement

This study was reviewed and approved by Ethics Committee of Zhongshan Hospital, Fudan University (CR2022-018). The patients/participants provided their written informed consent to participate in this study. Written informed consent was obtained from the individual(s) for the publication of any potentially identifiable images or data included in this article.

## Author contributions

JD and TC treated the patient. AH wrote the manuscript. JD and TC reviewed the manuscript. All authors have contributed to the manuscript and approved the submitted version.

## Funding

This study was supported by the National Natural Science Foundation of China (Grant Number: 81972508).

## Conflict of interest

The authors declare that the research was conducted in the absence of any commercial or financial relationships that could be construed as a potential conflict of interest.

## Publisher’s note

All claims expressed in this article are solely those of the authors and do not necessarily represent those of their affiliated organizations, or those of the publisher, the editors and the reviewers. Any product that may be evaluated in this article, or claim that may be made by its manufacturer, is not guaranteed or endorsed by the publisher.

## References

[1] Monda L Warnke R Rosai J. A primary lymph node malignancy with features suggestive of dendritic reticulum cell differentiation. A report of 4 cases. Am J Pathol (1986) 122(3):562–72. PMC1888214 2420185

[2] Chen T Gopal P. Follicular dendritic cell sarcoma. Arch Pathol Lab Med (2017) 141(4):596–9. doi: 10.5858/arpa.2016-0126-RS 28353378

[3] Pang J Mydlarz WK Gooi Z Waters KM Bishop J Sciubba JJ. Follicular dendritic cell sarcoma of the head and neck: Case report, literature review, and pooled analysis of 97 cases. Head Neck (2016) 38 Suppl 1:E2241–9. doi: 10.1002/hed.24115 PMC4696909 25917851

[4] Kaur R Mehta J Borges A. Extranodal follicular dendritic cell sarcoma-a review: "What the mind does not know the eye does not see". Adv Anat Pathol (2021) 28(1):21–9. doi: 10.1097/PAP.0000000000000281 32991350

[5] Jain P Milgrom SA Patel KP Nastoupil L Fayad L Wang M. Characteristics, management, and outcomes of patients with follicular dendritic cell sarcoma. Br J Haematol (2017) 178(3):403–12. doi: 10.1111/bjh.14672 PMC5903684 28382648

[6] Gounder M Desai V Kuk D Agaram N Arcila M Durham B. Impact of surgery, radiation and systemic therapy on the outcomes of patients with dendritic cell and histiocytic sarcomas. Eur J Cancer (2015) 51(16):2413–22. doi: 10.1016/j.ejca.2015.06.109 PMC5087129 26298731

[7] Saygin C Uzunaslan D Ozguroglu M Senocak M Tuzuner N. Dendritic cell sarcoma: A pooled analysis including 462 cases with presentation of our case series. Crit Rev Oncol Hematol (2013) 88(2):253–71. doi: 10.1016/j.critrevonc.2013.05.006 23755890

[8] Lee MY Bernabe-Ramirez C Ramirez DC Maki RG. Follicular dendritic cell sarcoma and its response to immune checkpoint inhibitors nivolumab and ipilimumab. BMJ Case Rep (2020) 13(4):e234363. doi: 10.1136/bcr-2020-234363 PMC7202776 32327462

[9] Shah P Shah S Agostino N. Disease response to pazopanib in follicular dendritic cell sarcoma. Case Rep Oncol (2020) 13(3):1131–5. doi: 10.1159/000509771 PMC7548949 33082759

[10] Li H Li J Wang X Pang H Di Y Ren G. Promising clinical outcome with long term follow-up after body gamma knife stereotactic radiosurgery for patients with early stage non-small cell lung cancer. Front Oncol (2018) 8:618. doi: 10.3389/fonc.2018.00618 30622929 PMC6308148

[11] Cao Y Zhang J Li T Qiu J Zhang L Zhuang Y. Comparison of dose distributions among five radiotherapy apparatuses in stereotactic body radiation therapy for pancreatic cancer. Chin J Radiat Oncol (2021) 30(2):156–63. doi: 10.3760/cma.j.cn113030-20190606-00214

[12] Wei J Dong X Du F Tang S Wei H. Successful gamma knife radiosurgery combined with s-1 in an elderly man with local recurrent pancreatic cancer: A case report. Med (Baltimore) (2017) 96(51):e9338. doi: 10.1097/MD.0000000000009338 PMC5758216 29390514

[13] Yu W Tang L Lin F Li D Wang J Yang Y. Stereotactic radiosurgery, a potential alternative treatment for pulmonary metastases from osteosarcoma. Int J Oncol (2014) 44(4):1091–8. doi: 10.3892/ijo.2014.2295 PMC3977803 24535005

[14] Li J Zhou ML Zhou SH. Clinical and pathological features of head and neck follicular dendritic cell sarcoma. Hematology (2015) 20(10):571–83. doi: 10.1179/1607845415Y.0000000008 25831474

[15] Wu YL Wu F Xu CP Chen GL Zhang Y Chen W. Mediastinal follicular dendritic cell sarcoma: A rare, potentially under-recognized, and often misdiagnosed disease. Diagn Pathol (2019) 14(1):5. doi: 10.1186/s13000-019-0779-3 30646936 PMC6334468

[16] Lopez-Hisijos N Omman R Pambuccian S Mirza K. Follicular dendritic cell sarcoma or not? a series of 5 diagnostically challenging cases. Clin Med Insights Oncol (2019) 13:2013427021. doi: 10.1177/1179554919844531 PMC6537047 31205436

[17] Ang WW Bundele MM Shelat VG. Follicular dendritic cell sarcoma: Rare presentation of incidental large hepatic mass. Ann Hepatobiliary Pancreat Surg (2019) 23(1):74–6. doi: 10.14701/ahbps.2019.23.1.74 PMC6405365 30863812

[18] Sabattini E Bacci F Sagramoso C Pileri SA. WHO classification of tumours of haematopoietic and lymphoid tissues in 2008: An overview. Pathologica (2010) 102(3):83–7. 21171509

[19] Bai LY Kwang WK Chiang IP Chen PM. Follicular dendritic cell tumor of the liver associated with Epstein-Barr virus. Jpn J Clin Oncol (2006) 36(4):249–53. doi: 10.1093/jjco/hyl001 16533803

[20] Chan JK Fletcher CD Nayler SJ Cooper K. Follicular dendritic cell sarcoma. Clinicopathologic analysis of 17 cases suggesting a malignant potential higher than currently recognized. Cancer-Am Cancer Soc (1997) 79(2):294–313. 9010103

[21] Vermi W Lonardi S Bosisio D Uguccioni M Danelon G Pileri S. Identification of CXCL13 as a new marker for follicular dendritic cell sarcoma. J Pathol (2008) 216(3):356–64. doi: 10.1002/path.2420 18792075

[22] Grogg KL Macon WR Kurtin PJ Nascimento AG. A survey of clusterin and fascin expression in sarcomas and spindle cell neoplasms: Strong clusterin immunostaining is highly specific for follicular dendritic cell tumor. Mod Pathol (2005) 18(2):260–6. doi: 10.1038/modpathol.3800294 15467709

[23] De Pas T Spitaleri G Pruneri G Curigliano G Noberasco C Luini A. Dendritic cell sarcoma: An analytic overview of the literature and presentation of original five cases. Crit Rev Oncol Hematol (2008) 65(1):1–7. doi: 10.1016/j.critrevonc.2007.06.003 17658269

[24] Facchetti F Lorenzi L. Follicular dendritic cells and related sarcoma. Semin Diagn Pathol (2016) 33(5):262–76. doi: 10.1053/j.semdp.2016.05.002 27318412

[25] Conry RM. Response of follicular dendritic cell sarcoma to gemcitabine and docetaxel: report of two cases and literature review. Clin Sarcoma Res (2014) 4:6. doi: 10.1186/2045-3329-4-6 25009738 PMC4089565

[26] Gupta AM Goel M Sahay A Janjal SP Patkar S. Role of adjuvant chemotherapy in extranodal follicular dendritic cell sarcoma. ACG Case Rep J (2019) 6(3):1–4. doi: 10.14309/crj.0000000000000008 PMC6658018 31620492

[27] Lei Y Zhao S Jiang M. Unexpected favorable outcome to PD-1 antibody plus lenvatinib in a patient with recurrent intestinal follicular dendritic cell sarcoma: A case report and literature review. Front Immunol (2021) 12:653319. doi: 10.3389/fimmu.2021.653319 34566950 PMC8456086

[28] Rothrock R Pennington Z Ehresman J Bilsky MH Barzilai O Szerlip NJ. Hybrid therapy for spinal metastases. Neurosurg Clin N Am (2020) 31(2):191–200. doi: 10.1016/j.nec.2019.11.001 32147010

[29] Jaffray DA. Image-guided radiotherapy: From current concept to future perspectives. Nat Rev Clin Oncol (2012) 9(12):688–99. doi: 10.1038/nrclinonc.2012.194 23165124

[30] Xia T Li H Sun Q Wang Y Fan N Yu Y. Promising clinical outcome of stereotactic body radiation therapy for patients with inoperable stage I/II non-small-cell lung cancer. Int J Radiat Oncol Biol Phys (2006) 66(1):117–25. doi: 10.1016/j.ijrobp.2006.04.013 16765528

[31] Jones B Dale RG Deehan C Hopkins KI Morgan DA. The role of biologically effective dose (BED) in clinical oncology. Clin Oncol (R Coll Radiol) (2001) 13(2):71–81. doi: 10.1053/clon.2001.9221 11373882

