# Supplementary material for: Promising clinical outcome after body gamma knife radiotherapy for mediastinal follicular dendritic cell sarcoma with thoracic spine invasion and iliac metastasis: A case report and literature review
Source: Front Oncol. 2022 Sep 16;12:919644. doi: 10.3389/fonc.2022.919644 (PMC9524614; doi:10.3389/fonc.2022.919644)
Supplement: Supplementary file 1 [file DataSheet_1.docx]

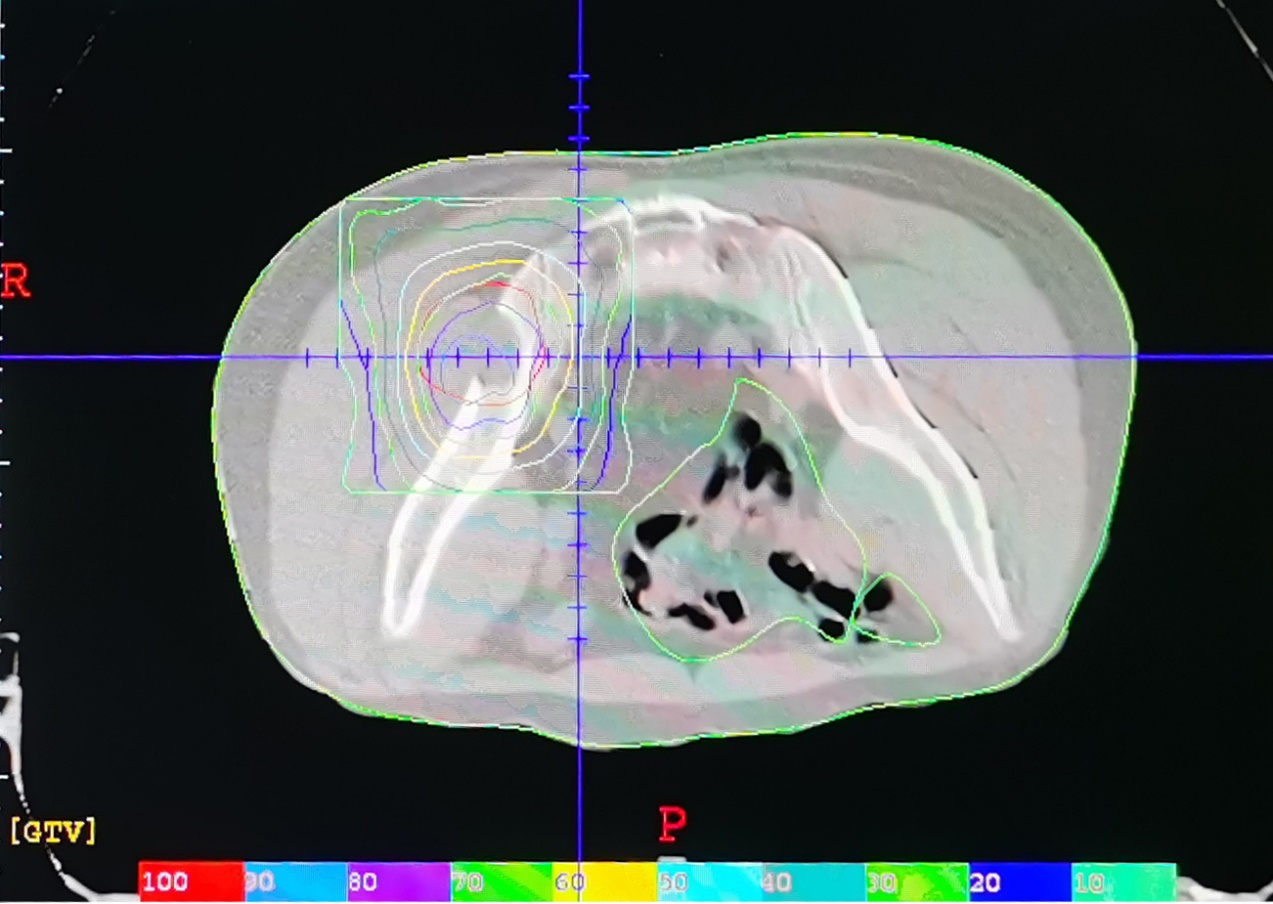


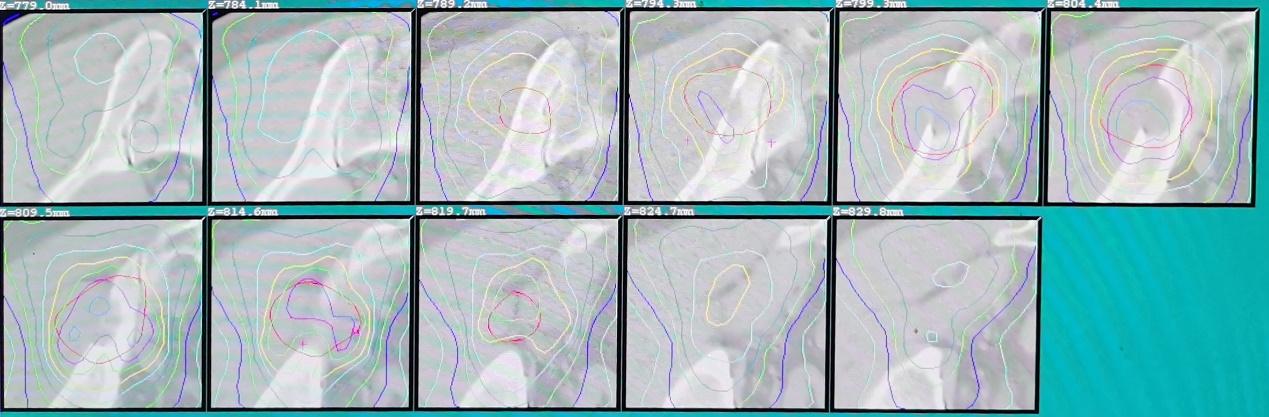


Supplementary Figure 1 Radiotherapy target planning of iliac lesion, different colors represent different isodose lines


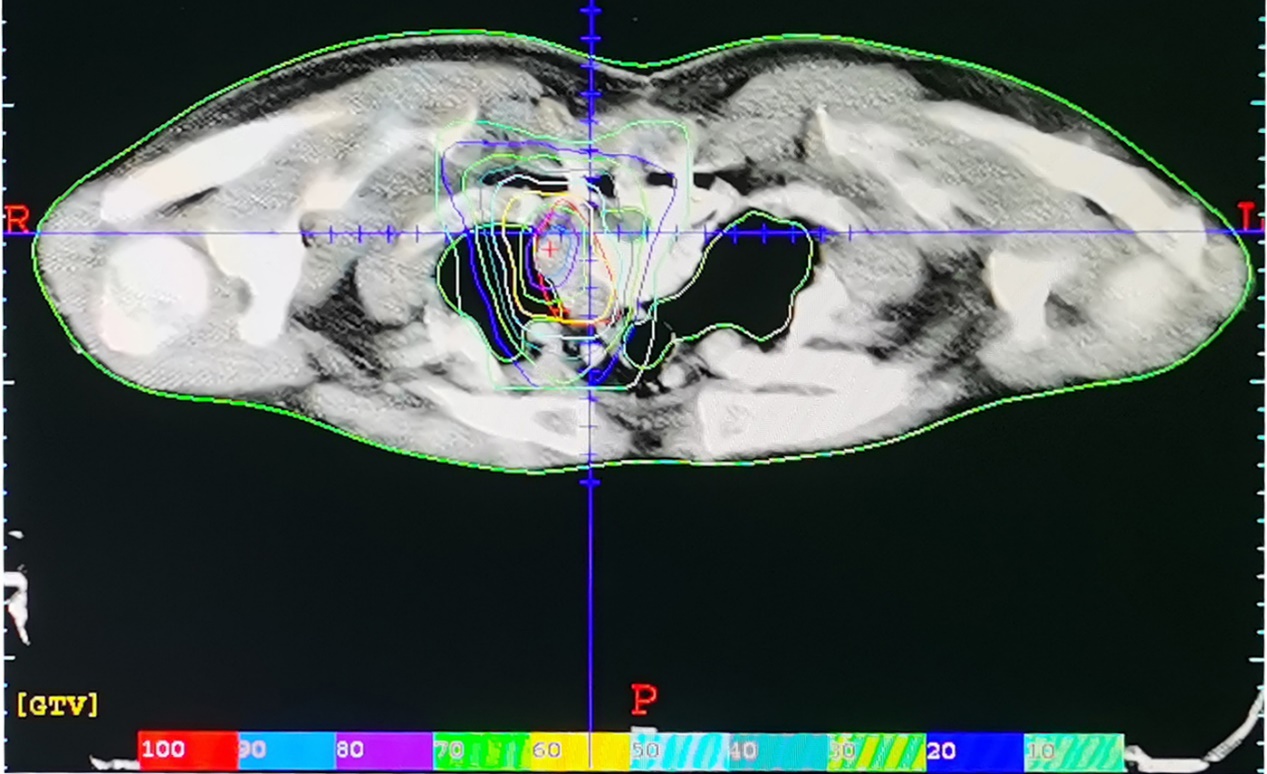


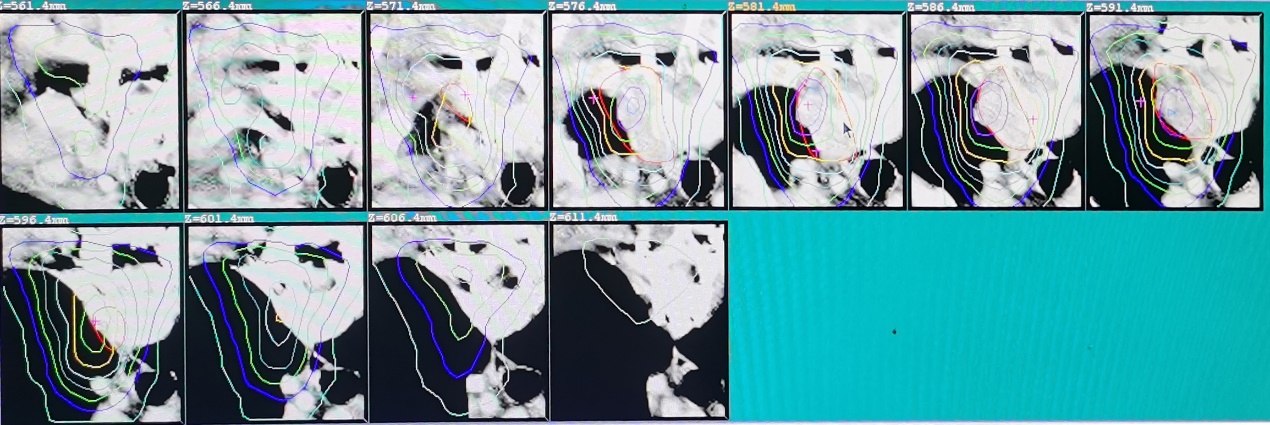


Supplementary Figures 2 Radiotherapy target planning of thoracic lesion, different colors represent different isodose lines
